# Efficacy and safety of the eight-chop technique in phacoemulsification for patients with cataract

**DOI:** 10.1097/j.jcrs.0000000000001141

**Published:** 2023-01-10

**Authors:** Tsuyoshi Sato

**Affiliations:** From the Department of Ophthalmology, Sato Eye Clinic, Chiba-ken, Japan.

## Abstract

The aim of this study was to estimate the efficacy of the 8-chop technique in phacoemulsification surgeries of patients with cataract.

**Setting:**

Sato Eye Clinic, Chiba-ken, Japan.

**Design:**

Prospective study.

**Methods:**

Patients were classified into 3 groups (Grade II, Grade III, and Grade IV; n = 50 each) according to the firmness of their lens nuclei. The Eight-chopper I was used for Grade II, Eight-chopper II for Grade III, and Lance-chopper for Grade IV. The best-corrected visual acuity, intraocular pressure (IOP), and endothelial cell density were evaluated at 7 and 19 weeks postoperatively. The primary outcome measures were the mean operative time, mean phaco time, cumulative dissipated energy (CDE), and volume of fluid used.

**Results:**

150 cataract surgeries were performed. The operative time (minutes), phaco time (seconds), CDE, and volume of fluid used (milliliters) differed significantly among the 3 groups, increasing in the following order: Grade II, Grade III, and Grade IV (*P* < .01). The corneal endothelial cell density did not decrease significantly in the 3 groups at 19 weeks postoperatively (*P* = .09). The rate of endothelial cell loss was 0.9% ± 5.9%, 1.0% ± 10.3%, and 5.3% ± 11.1% in the Grade II, III, and IV groups at 19 weeks postoperatively, respectively. There were significant reductions in the IOP at 7 and 19 weeks postoperatively compared with the preoperative IOP in the 3 groups (*P* < .01).

**Conclusions:**

The 8-chop technique was effective and safe in phacoemulsification for patients with cataracts with lens nuclei of varying hardness.

## Abstract 2

The eight-chop technique, which is characterized by the division of the lens nucleus into 8 segments, is effective and safe in phacoemulsification for patients with cataract.

Phacoemulsification began with the single-handed engraving technique and evolved into the divide-and-conquer technique by Gimbel in 1991, the phaco-chop technique by Nagahara in 1993, and the prechop technique by Akahoshi in 1994.^1–3^ Many other variations of phacoemulsification techniques have been described.^4–8^ The use of ultrasonic oscillation energy increases the risk for injury to the corneal endothelial cells.^9^ Therefore, all these techniques are intended to decrease the total ultrasound time and energy used during nucleus emulsification and reduce the stress on the zonular fibers and capsule.

Akahoshi first developed the prechop technique in 1992 and presented it at the American Society of Cataract and Refractive Surgery Film Festival in 1994.^10^ The prechop technique manually divides the nucleus under a ophthalmic viscosurgical device before phacoemulsification.^10^ Compared with conventional grooving, divide-and-conquer, and phaco-chop techniques, the total ultrasound energy is drastically reduced, and the aspiration time and volume of the fluid used are significantly lower. However, the prechop technique is extremely difficult to perform and is rarely used globally. The prechopper tip is large and difficult to manipulate in the anterior chamber and is also dull, making it difficult to insert into the lens nucleus. In addition, the thick tip of the prechopper adds to the difficulty of insertion into the lens nucleus because of its resistance after insertion. This may be because only 4 articles, to the author's knowledge, have been published on the prechop technique in the past 30 years, with the exception of the study by Akahoshi.^11–14^

I have been using the prechop technique in cataract surgeries since 2000, and I developed the 8-chop technique in 2002. This is characterized by the division of the lens nucleus into 8 segments, instead of only 4 as in the prechop technique. I presented the 8-chop technique at the 32nd Annual Meeting of the Japanese Society of Ophthalmic Surgery in 2009.^15^ The Lance-chop technique is an 8-chop technique that uses the Lance-chopper and nucleus sustainer for cases with a hard lens nucleus. Since the Eight-chopper I and the Eight-chopper II cannot be inserted into a hard lens, the Lance-chop technique is used to divide the lens nucleus by inserting the Lance-chopper into the lens nucleus while supporting the lens equator with the nucleus sustainer and avoiding stress on zonular fibers. I presented this at the 42nd Annual Meeting of the Japanese Society of Ophthalmic Surgery in 2019.^16^

The aim of this study was to determine the operative time, phaco time, cumulative dissipated energy (CDE), and volume of the fluid used in the 8-chop technique and confirm its superiority over previous techniques. If the 8-chop technique, which is an improved version of the prechop technique, is found to be less invasive, it may become the technique of choice for more cataract surgeons.

## METHODS

In this study, the efficiency and safety of the 8-chop technique were estimated. This study comprised eyes of patients with cataracts who had undergone phacoemulsification and posterior chamber intraocular lens (IOL) implantation between June 2018 and March 2022. Patients who had visited the clinic with a diagnosis of cataracts were enrolled in the study. The exclusion criteria were corneal disease or opacity, uveitis, pupillary dilation problem, and previous trauma or surgery. The data that support the findings of this study are available on request from the corresponding author.

The study protocol adhered to the tenets of the Declaration of Helsinki and was approved by the review board. Informed consent for participation in this study was obtained from each patient. Preoperatively, all patients underwent slitlamp and retinal examinations, and their corrected distance visual acuity (CDVA) and intraocular pressure (IOP) were measured. Endothelial cell density (cells/mm^2^) was measured using a noncontact specular microscope (EM-3000, Topcon Corp.). The firmness of the nucleus was graded using the Emery classification, based on which the patients were classified into 1 of 3 groups (Grade II, Grade III, or Grade IV).^17^ As an exception, the Grade IV group included Grade IV and V cataracts. Phacoemulsification was performed by the same surgeon, who was experienced in the 8-chop technique, using the Centurion phacoemulsification unit (Alcon Laboratories, Inc.).

**Figure 1.**
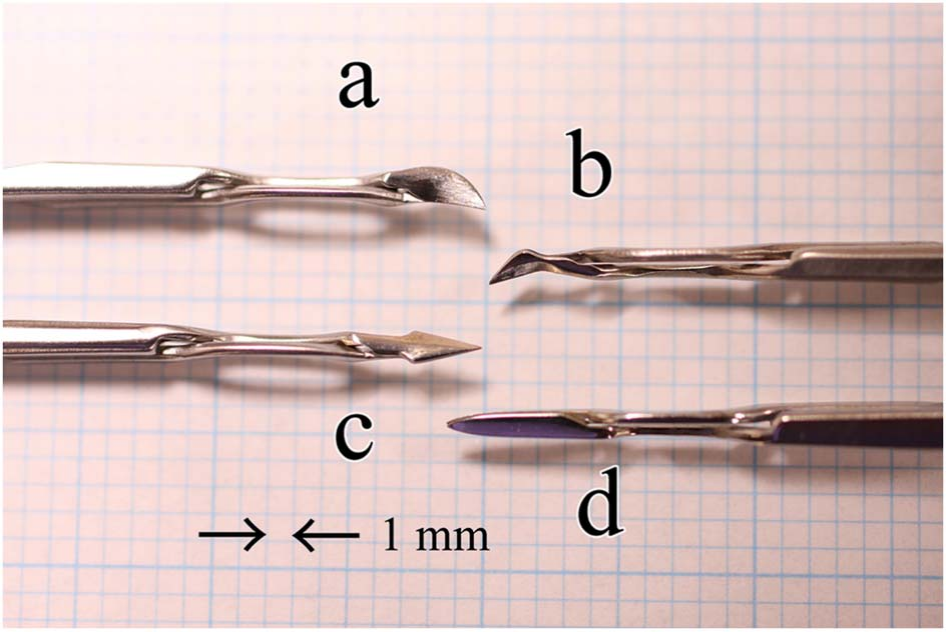
Eight-choppers and Universal II prechopper. *a*: the Eight-chopper I (SP-8193); *b*: the Eight-chopper II (SP-8402); *c*: the Lance-chopper (SP-9989); and *d*: The Universal II Prechopper (AE-4192).

Three new surgical instruments were designed and developed to perform the 8-chop technique (Figure 1). The research team designed these eight-choppers and requested a manufacturing company to produce them. The Eight-chopper I (SP-8193, ASICO LLC) has a smaller tip than that of the conventional prechopper, with a length and width of 3.2 mm and 1.4 mm, respectively, as well as a sharper leading edge, and was used for the Grade II group. The Eight-chopper II (SP-8402, ASICO LLC) has a smaller tip (2.5 mm long and 0.8 mm wide) that is angled so that it can be inserted vertically into the lens nucleus for the Grade III group. For the Grade IV group, the tip of the Lance-chopper (SP-9989, ASICO LLC) was 3.0 mm long and 1.3 mm wide, and the leading edge was sharper.

**Figure 2.**
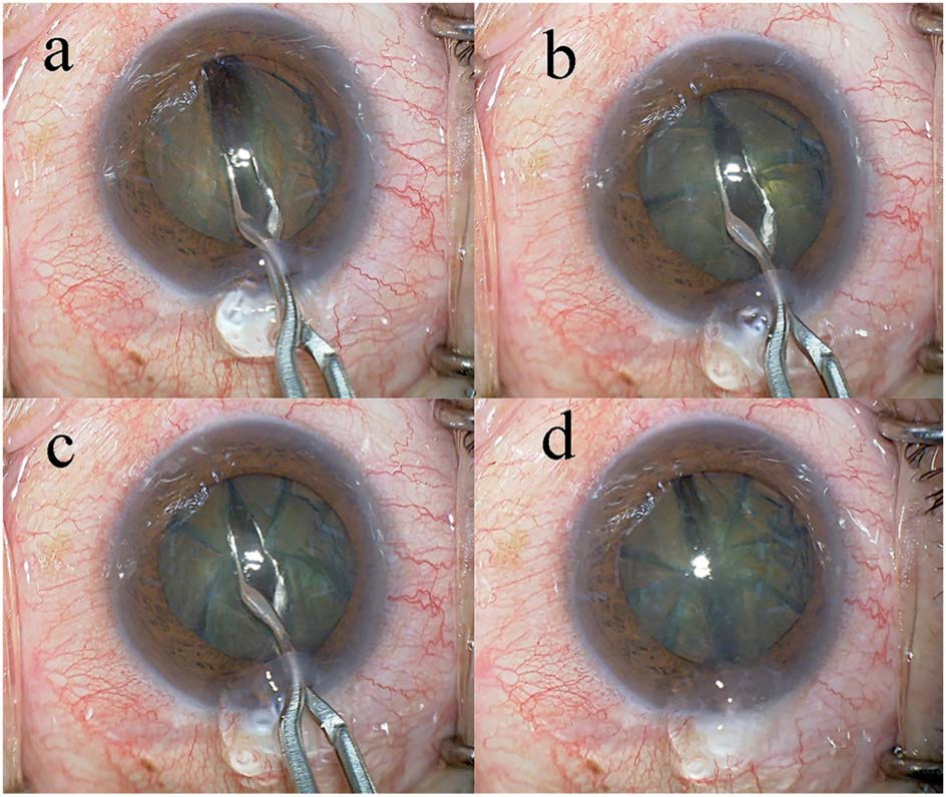
*a*: First, the lens nucleus is divided into hemispheres using the Eight-chopper. *b*: Then, the lens nucleus is rotated 90 degrees and divided into quadrants. *c*: The lens nucleus divided into quadrants is rotated 45 degrees and divided into 6 segments. *d*: Finally, the remaining quadrants of the lens nucleus are also divided to complete the eight segmentation.

**Figure 3.**
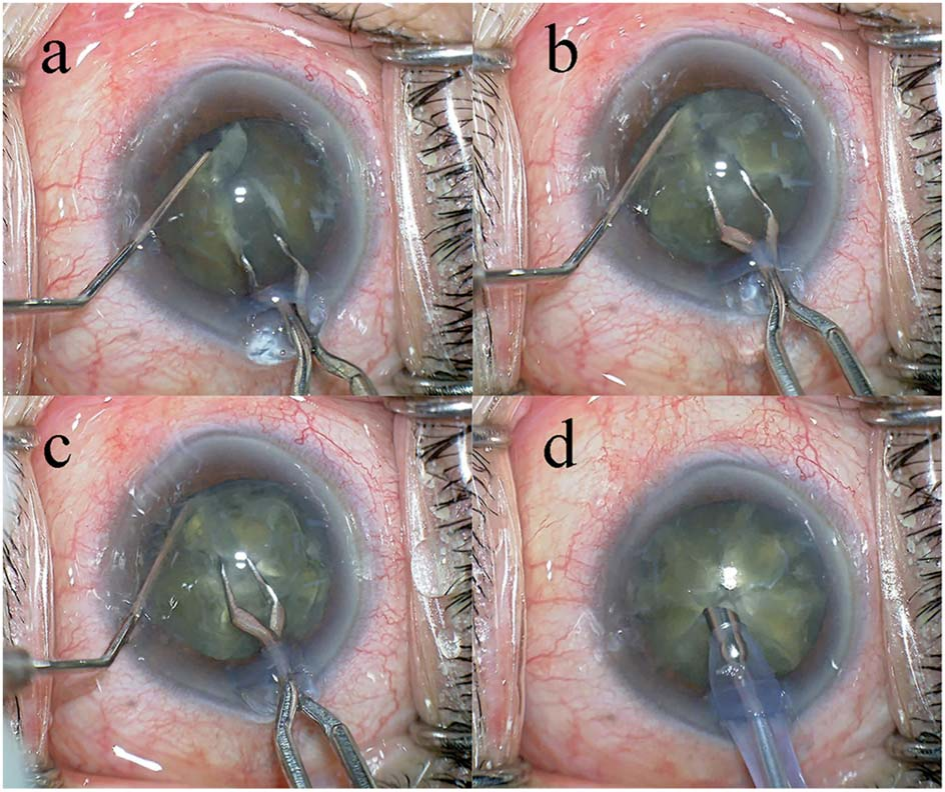
*a*: First, the lens nucleus is divided into hemispheres using the Lance-chopper, while the nucleus sustainer (AE-2530) is inserted through the side port to support the equator of the lens nucleus. *b*: Then, the lens nucleus is rotated 90 degrees and divided into quadrants. *c*: The lens nucleus divided into quadrants is rotated 45 degrees and divided into 6 segments. *d*: Finally, the remaining quarter of the lens nucleus is also divided to complete the eight segmentation.

In all surgeries, a temporal, clear, corneal incision was formed using a 3.0 mm steel keratome. After the injection of sodium hyaluronate into the anterior chamber, a 6.2 to 6.5 mm continuous curvilinear capsulorrhexis (CCC) was created with a capsule forceps. The soft-shell technique was used in the Grade III and IV groups.^18^ Brilliant blue G (0.025%) was used to improve visualization of the capsule in cases with dense cataracts or corneal opacity. Hydrodissection was performed with a 27-gauge cannula; however, hydrodelineation was never performed. The lens nucleus was cracked into 8 segments using the Eight-chopper I in the Grade II group, the Eight-chopper II in the Grade III group, and the Lance-chopper in the Grade IV group (Figures 2 and 3; Videos 1 and 2 available at http://links.lww.com/JRS/A801 and http://links.lww.com/JRS/A802). A 1 side-port incision was made using the 23-gauge microvitreoretinal knife 90 degrees from the main incision in the Lance-chop technique. The 8 segments were phacoemulsified and aspirated at the depth of the iris plane. The capsular bag was aspirated with the irrigation/aspiration tip to remove the cortical material. A ophthalmic viscosurgical device was injected, and a foldable IOL (Alcon Laboratories, Inc.) with polymethyl methacrylate haptics was inserted in the capsular bag with an injector system. The ophthalmic viscosurgical device was then aspirated. The Centurion phacoemulsification unit was used in all cases and had a flow rate of 32 mL/min, a maximum ultrasound power of 80%, and a 1.1 mm tip. The wound was sealed using stromal hydration if necessary. At the end of the operation, the anterior chamber was replaced by balanced salt solution containing moxifloxacin (0.5 mg/mL).

**Video 1:** The eight segmentation of the lens nucleus using the Eight-chopper II.

**Video 2:** The eight segmentation of the lens nucleus using the Lance chopper and the nucleus sustainer.

The intraoperative outcome measures were phaco time (seconds), CDE, operative time (minutes), volume of fluid used (milliliters), and the rate of intraoperative complications. The operative time was measured from the beginning of the corneal incision to the end of ophthalmic viscosurgical device aspiration. Patients were followed up on postoperative days 1 and 2 and at postoperative weeks 1, 3, 7, and 19. The postoperative outcome measures were CDVA, IOP measurements, and endothelial cell density (cells/mm^2^). Data on the outcome measures at 7 and 19 weeks postoperatively were used to conduct this study.

Statistical analyses were performed to compare the results between the groups using either 1-way analysis of variance (ANOVA) or 1-way ANOVA followed by the Bonferroni post hoc test using Excel Toukei (v. 7.0, Esumi Co. Ltd.). The preoperative and postoperative results of the 3 groups were analyzed using 2-way ANOVA using Excel Toukei (v. 7.0). The variance of each group was confirmed to be equally distributed using the Bartlett test. *P* < .05 was considered statistically significant.

## RESULTS

This study comprised 150 eyes of 107 patients with cataract who had undergone phacoemulsification and posterior chamber IOL implantation. Each cataract density group (Grade II, III, and IV) included 50 eyes. A total of 113 eyes could be followed up to 19 weeks postoperatively: 34 in the Grade II group, 42 in the Grade III group, and 37 in the Grade IV group. The patients' characteristics and intraoperative parameters are given in Table 1. There were significant differences in the mean ages among the groups (*P* < .01, 1-way ANOVA). The mean operative time in the Grade II group was significantly shorter than that in the Grade III and IV groups (*P* < .01, 1-way ANOVA followed by the Bonferroni post hoc test). The mean operative time in the Grade III group was also significantly shorter than that in the Grade IV group (*P* < .01, 1-way ANOVA followed by the Bonferroni post hoc test). The mean phaco time in the Grade II group was significantly shorter than that in the Grade III and IV groups (*P* < .01, 1-way ANOVA followed by the Bonferroni post hoc test). The mean phaco time in the Grade III group was also significantly shorter than that in the Grade IV group (*P* < .01, 1-way ANOVA followed by the Bonferroni post hoc test). The CDE in the Grade II group was significantly lesser than that in the Grade III and IV groups (*P* < .01, 1-way ANOVA followed by the Bonferroni post hoc test). The CDE in the Grade III group was also significantly lesser than that in the Grade IV group (*P* < .01, 1-way ANOVA followed by the Bonferroni post hoc test). The volume of the fluid used in the Grade II group was significantly lesser than that used in the Grade III and IV groups (*P* < .01, 1-way ANOVA followed by the Bonferroni post hoc test). The volume of the fluid used in the Grade III group was also significantly lesser than that used in the Grade IV group (*P* < .01, 1-way ANOVA followed by the Bonferroni post hoc test).

**Table 1.** Preoperative characteristics and intraoperative parameters

Parameters	Grade II	Grade III	Grade IV	*P* value
n	50	50	50	
Age (y), mean ± SD	72.8 ± 5.6	77.5 ± 7.2	77.1 ± 8.8	<.01*
Sex, n (%)				
M/F	19 (38)/31 (62)	16 (32)/34 (68)	29 (58)/21 (42)	
Operative time (min), mean ± SD	3.72 ± 0.45	5.42 ± 1.05	9.63 ± 2.15	<.01^*^
Phaco time (s), mean ± SD	11.6 ± 4.1	20.2 ± 5.4	28.7 ± 8.5	<.01^*^
CDE, mean ± SD	5.00 ± 1.88	9.24 ± 2.00	14.81 ± 5.20	<.01^*^
Volume of fluid used (mL), mean ± SD	22.9 ± 6.1	33.3 ± 6.5	44.1 ± 8.4	<.01^*^

CDE = cumulative dissipated energy

* Statistically significant (1-way ANOVA)

The CDVA (logMAR) preoperatively, at 7 weeks, and 19 weeks postoperatively in the Grade II group were 0.081 ± 0.13 (mean ± SD), −0.054 ± 0.043, and −0.048 ± 0.047, respectively. The CDVA preoperatively, at 7 weeks, and 19 weeks postoperatively in the Grade III group were 0.30 ± 0.34, −0.024 ± 0.095, and −0.023 ± 0.096, respectively. The CDVA preoperatively, at 7 weeks, and 19 weeks postoperatively in the Grade IV group were 0.52 ± 0.58, −0.039 ± 0.22, and 0.027 ± 0.23, respectively. There were significant differences in the CDVA among the groups preoperatively, at 7 weeks, and 19 weeks postoperatively (*P* < .01, *P* < .01, and *P* = .038, respectively, 1-way ANOVA). The CDVA differed significantly between the groups preoperatively and at 7 weeks postoperatively (*P* < .01, 1-way ANOVA). The CDVA between the groups also differed significantly preoperatively and at 19 weeks postoperatively (*P* < .01, 1-way ANOVA).

The changes in the corneal endothelial cell density in each group are given in Table 2. The preoperative corneal endothelial cell density did not differ significantly among the groups (*P* = .30, 1-way ANOVA) nor did it differ at 7 and 19 weeks postoperatively (*P* = .60, *P* = .18, respectively, 1-way ANOVA). Furthermore, the corneal endothelial density did not differ significantly among the 3 groups, even at 7 and 19 weeks postoperatively (*P* = .22, *P* = .09, respectively, 2-way ANOVA).

**Table 2.** Preoperative and postoperative corneal endothelial cell density measurements (cells/mm^2^)

Parameters	Grade II	Grade III	Grade IV	*P* value
Preop	2579 ± 234	2502 ± 290	2565 ± 260	.30
7-wk postop	2456 ± 358	2390 ± 392	2448 ± 303	.60 .22
Cell loss (%)	1.0 ± 4.4	1.1 ± 7.8	3.2 ± 8.3	
19-wk postop	2555 ± 259	2450 ± 378	2444 ± 355	.18 .09
Cell loss (%)	0.9 ± 5.9	1.0 ± 10.3	5.3 ± 11.1	

The changes in the IOP of each group are given in Table 3. There were no significant differences in the preoperative IOP among the groups (*P* = .19, 1-way ANOVA). The IOP was significantly lower at 7 and 19 weeks postoperatively than the preoperative IOP in all 3 groups (*P* < .01, *P* < .01, respectively, 2-way ANOVA). The IOPs of the 3 groups did not differ significantly at 7 and 19 weeks postoperatively (*P* = .83, *P* = .69, respectively, 1-way ANOVA). No intraoperative complications were observed, and no capsulorrhexis tears occurred in the 3 groups.

**Table 3.** Preoperative and postoperative IOP measurements (mm Hg)

Parameters	Grade II	Grade III	Grade IV	*P* value
Preop	13.9 ± 2.0	14.6 ± 1.9	14.4 ± 2.1	.19
7-wk postop	12.3 ± 2.3	12.1 ± 2.1	12.1 ± 2.3	.83^*^
19-wk postop	11.7 ± 2.5	12.0 ± 2.0	11.9 ± 2.2	.69^*^

* Statistically significant *P* <.01 (2-way ANOVA)

## DISCUSSION

This study revealed that the operative time of the eight-chop technique was 4 to 6 minutes in the Grade II and III groups, which is extremely short compared with that of other techniques that have been reported to take 10 to 19 minutes.^18–24^ Even in cases with hard lens nuclei, the Lance-chop technique can be completed in less than 10 minutes, which may be shorter than the phaco-chop technique, which reportedly takes 12 minutes.^25^ The 8-chop technique had a lower phaco time and CDE than other techniques; it also had an extremely low volume of fluid, that is, one-third to one-fourth of that used for other techniques.^18–21,23–26^ In particular, the use of a low volume of fluid means that ultrasound and irrigation/aspiration tips are inserted into the eye for a short period, which may have little impact on ocular tissues other than corneal endothelial cells.

Corneal endothelial cell density represents the true summation of intraocular insult during surgery, and its assessment is the key to its comparison between various techniques.^27^ Previous studies have reported a 5% to 16% decrease in corneal endothelial cell density after cataract surgery in the first few postoperative months; however, in this study, the decrease was 0.9%, 1.0%, and 5.3% in the Grade II, III, and IV groups, respectively, with no significant difference after 19 weeks in all groups.^18,19,21,24–26,28^ These results indicate that the 8-chop technique may be advantageous in surgical invasion of the intraocular tissue.

A decrease in the IOP after cataract surgery has been reported in both nonglaucoma and glaucoma patients.^29–32^ Similarly, in this study using the 8-chop technique, the postoperative IOP decreased by 2.2 to 2.5 mm Hg in all 3 groups. By comparing the 8-chop technique with other techniques, it may become clear whether the 8-chop technique is effective in IOP reduction for cataract patients with concomitant glaucoma.

There are a few differences between the prechop and 8-chop techniques. First, in the prechop technique, the lens nucleus is usually divided into 4 sections, whereas in the 8-chop technique, the lens nucleus is always divided into 8 sections.^10,33^ This is the advantage of the 8-chop technique over the prechop technique because the smaller divided pieces of the lens nucleus allow for more efficient phacoemulsification and aspiration.

The second difference is the development and use of special choppers with sharper and more delicate tips, such as the Eight-chopper 1, Eight-chopper 2, and Lance-chopper. These surgical instruments facilitate the eight-piece division of the lens nucleus, which is very difficult with the surgical instruments used in the prechop technique. Furthermore, these instruments are very useful in difficult cases because they reduce stress on the zonular fibers and lens capsule.

Third, in the 8-chop technique, the CCC is enlarged for safety. In the prechop technique, a CCC diameter of just <5 mm is recommended for a 5.5 mm diameter IOL and a CCC with a diameter of 5 mm is ideal for a 6 mm diameter IOL.^10,33^ In the 8-chop technique, a 6 mm diameter IOL is used and the CCC diameter is 6.2 to 6.5 mm. Creating a larger CCC reduces friction between the lens capsule and lens nucleus and facilitates rotation of the lens nucleus. In addition, phacoemulsification of the lens nucleus can be performed safely because it is not obstructed by the anterior lens capsule. Similarly, the lens cortex can also be aspirated safely and efficiently. The other major problem with small-radius CCCs is the development of capsule contraction syndrome. The radius of the CCC shrinks to 86% after surgery.^34^ However, a large CCC can impair the shrink wrap effect on IOL and cause IOL to tilt. This tilt can have a greater impact on visual function in toric and presbyopia-correcting IOLs than in monofocal lenses, and one should be cautious in its use.

Fourth, the Lance-chop technique is more ideal than the counter prechop technique for difficult cases, such as those with a hard lens nucleus or loose zonular fibers because it minimizes the stress on the posterior capsule and zonular fibers. The counter prechop technique is also used for dense cataracts in the prechop technique; however, the Lance prechopper is smaller and sharper than the Akahoshi Universal II prechopper (AE4192, ASICO LLC), allowing the lens nucleus to be divided safely and efficiently.^10^ Furthermore, it is not possible to eliminate the stress on the posterior capsule and zonular fibers with the divide-and-conquer and phaco-chop techniques.

The prechop and 8-chop techniques have features not possessed by other techniques. The prechop and 8-chop techniques can distinguish both the division and phacoemulsification of the lens nucleus, allowing the surgeon to concentrate on each individual procedure. In other techniques, such as the divide-and-conquer and phaco-chop techniques, wherein lens nucleus division and phacoemulsification are performed simultaneously, the accuracy of each procedure may be compromised. If one procedure is given more attention, lens nucleus division and phacoemulsification and aspiration can be performed with more certainty.

Another advantage of both the prechop and 8-chop techniques is that the ultrasound handpiece is held with both hands, allowing for delicate manipulation. In the divide-and-conquer and phaco-chop techniques, a hook or phaco-chopper is held in one hand and the ultrasound handpiece in the other, making it less stable compared with handling it with both hands. However, the study has some limitations. Even with the 8-chop technique, cases with weak zonular fibers, corneal opacity, mature cataract, and small pupil require difficult procedures; thus, each case must be prepared for, and all possible precautions must be taken preoperatively.

The prechop technique has the potential to reduce operative time but is rarely used because of its difficulty. The 8-chop technique is a more advanced technique and has the advantages of the prechop technique while eliminating its difficulties. Many cataract surgeons have abandoned the use of the prechop technique because of its difficulty, and even today, most cataract surgeons use the divide-and-conquer technique. A recent study using the divide-and-conquer technique reported a mean operative time of 19 min, a volume of fluid used of 360 mL, and a decrease in the corneal endothelial cell count of 8.5% at 3 weeks postoperatively.^24^ In the future, the effectiveness of the eight-chop technique may be confirmed by comparing it with the divide-and-conquer and phaco-chop techniques.

A 3 mm corneal incision used in this study needs to be considered in induced astigmatism because the size of the incision in modern cataract surgery is usually around 2.4 mm. The Eight-choppers used in this study can also be used with a corneal incision width of 2.6 mm, so it is possible to perform the 8-chop technique with a smaller corneal incision width.

In conclusion, to our knowledge, this is the first study to report that the 8-chop technique, in which the lens nucleus is always divided into 8 sections before phacoemulsification and aspiration, followed by efficient processing of the lens nucleus, has a very short-operative time and less CDE and volume of fluid used, as well as minimal corneal endothelial cell loss. This study did not compare the results with the prechop, phaco-chop, or divide-and-conquer techniques, and this should be fully considered when evaluating the present results. However, many other studies have been conducted using the phaco-chop and divide-and-conquer techniques, and we believe it is possible to partially evaluate the safety and efficiency of the 8-chop technique by comparing it with those results. In particular, the Lance-chop technique can safely divide the hard lens without stressing the zonular fibers, making it possible to perform phacoemulsification surgery even in cases in which division of the lens nucleus is very difficult. If more surgeons are able to perform the Lance-chop technique, it may be possible to provide safer phacoemulsification surgery to patients who have previously undergone intracapsular or extracapsular cataract extraction because of difficulties.

**WHAT WAS KNOWN**

- Phacoemulsification began with the single-handed engraving technique and evolved into the divide-and-conquer, the phaco-chop, and the prechop technique.
- Previous studies have reported a 5% to 16% decrease in corneal endothelial cell density after cataract surgery.

**WHAT THIS PAPER ADDS**

- The eight-chop technique which divides the lens nucleus into 8 segments is safe and effective in phacoemulsification.
- The corneal endothelial cell density loss was 0.9% to 5.3%, with no significant difference after 19 weeks in this study.
- The Lance-chop technique is ideal technique for a hard lens nucleus or loose zonular fibers, as it minimizes the stress on the posterior capsule and zonular fibers.
